# Impact of Resource on Green Growth and Threshold Effect of International Trade Levels: Evidence from China

**DOI:** 10.3390/ijerph19052505

**Published:** 2022-02-22

**Authors:** Haiying Liu, Wenqi Guo, Yu Wang, Dianwu Wang

**Affiliations:** 1 School of Business and Management, Jilin University, Changchun 130012, China; liuhaiying@jlu.edu.cn; 2 School of Maritime Economics and Management, Dalian Maritime University, Dalian 116026, China; wdw20210051@dlmu.edu.cn; 3 Corporate Strategy Research Department, State Grid Energy Research Institute, Beijing 102211, China; wangyu34120513@163.com

**Keywords:** resource curse, green growth, import, export, panel threshold model

## Abstract

International trade levels can change the relationship between resource endowments and green economic growth. Therefore, this study tested the resource curse hypothesis from the perspective of green growth in China using provincial-level panel data for 2005–2017. Energy conservation and environmental improvement were considered under green growth to further analyze the regional mechanism of the resource curse. A panel threshold model was used to identify the impact of import and export threshold effects on the transformation of this mechanism. The resource curse hypothesis was found to be valid nationwide; it hindered green economic growth mainly by impeding energy conservation and curbing environmental improvement. In terms of regional differences in green growth, resource endowment had a positive impact on the eastern region, a negative impact on the central region, and no effect on the western region. When the levels of import and export trade exceeded the threshold values, the resource curse effect was enhanced by reducing energy conservation and weakened by promoting environmental improvement, respectively. Therefore, the Chinese government should establish a more reasonable import and export trade structure, promote changes to the energy structure and green technological innovation, and reduce the negative impact of resource endowment on green growth.

## 1. Introduction

Abundant natural resources are important sources of national economic growth as they are indispensable input factors for production. Simultaneously, the comparative advantages from resource endowments can considerably crowd out other input factors [1] by weakening the impetus for technological innovation [2] and hindering high-quality economic development; this is termed as the resource curse [3]. This phenomenon is reflected at the regional level in China, where Shanxi, Shaanxi, Inner Mongolia, and Heilongjiang are rich in mineral resources; however, in recent years, the economic development level of these provinces has remained lower than that of the southeast coastal regions [4]. Therefore, the relationship between resource abundance and economic development should be re-examined. Economic development does not only refer to an increase in the economic aggregate, but also to the content it encompasses that changes according to different stages of social development. Since joining the World Trade Organization (WTO), China has been committed to economic and trade exchanges with other countries, pursuing a high-growth economic development model and producing numerous low-tech value-added primary products. Moreover, some regions have rapidly developed large industries, forming several industrial clusters with high energy consumption and high pollution characteristics, and resulting in an increasingly severe short-board effect on resources and the environment. The Chinese government has gradually recognized the importance of conserving resources and protecting the environment, and introduced a series of policies to eliminate ineffective production capacity and optimize the industrial structure. From proposing that “clear waters and green mountains are as valuable as golden and silver mountains” to advocating the five major development concepts of innovation, coordination, green, openness, and sharing, green growth has become the theme of development at present. The traditional resource curse theory only considers the relationship between resource abundance and economic growth. However, the concept of growth defined by this theory is different from that of green growth in the current social context in China. Under the theme of green growth, if the relationship between resources and development can be interpreted from multiple dimensions, such as resource conservation, environmental protection, and economic growth, green total factor productivity (TFP) can be used to characterize the level of green growth and expand the conceptual scope of the resource curse theory. This would be more conducive to formulating and implementing various ecological and environmental policies.

Although the resource curse hypothesis remains unconfirmed, irrespective of whether it is true or false, the importance of resources for economic and social development cannot be neglected. Resource input is the starting point of production, and natural resources themselves cannot act as a “curse” on the development of human society, but must be a “blessing.” The resource curse is attributed to the scenario where economic development is excessively dependent on resources, and the production entities lack the incentive for technological innovation, resulting in a crowding-out effect on the other production factors. Owing to economic globalization, all countries participate in international trade with their respective advantages. For example, some countries in the Middle East and North Africa supply energy to the international market, and oil export trade increases the dependence of the economic development of the region on its resource endowments. However, an improved trade system can reduce the adverse impact of oil reserves on the performance of the real economy [5]. Import and export trade affect the input–output structure of resources; therefore, when the import and export levels are at different stages, their influence in changing the mechanism by which resource endowments affect green TFP should be explored. Therefore, we proposed the following hypothesis in this study: by adjusting the trade structure, the mechanism of the resource curse can be changed, and the negative impact of resource endowment on green growth can be reduced or even reversed. Thus, to test this hypothesis, we will investigate how the mechanism of the resource curse change under different levels of import and export trade, and through what route the changes occur.

In summary, this study first measures the green TFP of provinces in China through the super-efficiency data envelopment analysis (DEA) method and the Luenberger productivity index to characterize green growth, and uses a fixed-effect panel model to verify the existence and regional heterogeneity of the resource curse from the perspective of green growth. Then, green TFP is decomposed into energy conservation effect and environmental improvement effect to analyze the specific path of the impact of resource endowment on green growth. Finally, the threshold regression model is used to test whether international trade levels play a threshold role in the resource curse hypothesis, and through which route import and export trade levels may change the impact of resource endowments on green growth.

The study is structured as follows: Section 2 reviews the relevant literature on resource curse. Section 3 explains the adopted methods and data, including measurement and decomposition of green total factor productivity, fixed-effects model, and threshold regression model. Section 4 presents the results of models in Section 3, and their interpretation, and states the limitations of the study. Section 5 provides the main conclusions.

## 2. Literature Review

As a starting point of social production, natural resources should be a “blessing” for economic development [6,7]; however, at the national level, the economic growth performance of some resource-rich countries or regions is not outstanding, and even poor for some countries with scarce resources. Prebisch [8] first explored this distorted relationship between resource endowment and economic growth, and the resource curse hypothesis was formally proposed by Auty [3]. Many scholars have conducted extensive research on the existence of the resource curse hypothesis. Sachs et al. [9] conducted empirical studies using panel data from 95 countries between 1970 and 1990, and the results showed a negative correlation between resource abundance and economic growth. The SW model developed by them is known as the paradigm model of resource curse empirical research. They also found that in resource abundant countries, there is often a wage premium in the natural resource sector, crowding out entrepreneurial activity and curbing the country from upgrading its industrial structure, thus, inhibiting economic growth [10]. Numerous subsequent studies have also reached the same conclusion that the resource curse hypothesis holds at the national level [11,12,13]. Some scholars in China have confirmed the existence of a resource curse at the provincial or prefecture level in China, such as Xinjiang, Shanxi, and Inner Mongolia, where resource-rich provinces fall into resource traps [14,15,16,17,18,19,20]. Nevertheless, various studies have opposed the resource curse hypothesis [21]. Fang et al. [22] and Jing [23] conducted empirical tests using prefecture-level and provincial-level data in China, respectively, and did not observe any significant negative correlation between resource endowment and economic growth. In addition, based on Kuznets theory [24], various scholars have proposed that there is a nonlinear relationship between resource dependence and economic development [25,26].

Economic development is not the only criterion for evaluating social progress, and in the context of the country’s active advocacy toward sustainable development, the quality of the ecological environment has also become an important indicator to measure economic growth. In recent years, numerous studies have expanded the theoretical scope of the resource curse and further explored the relationship between resources and development from the perspective of green growth [27,28,29]. Because green TFP can evaluate the quality of economic development based on resource input, environmental pollution, and economic growth, it is widely used in empirical research. Shao et al. [27] proposed the “conditional resource curse” hypothesis and reported that the dependence of the resource industry shows an inverted U-shaped curve relationship for both economic growth and green TFP growth. Li and Xu [28] used the nonradial directional distance function to measure green TFP in 275 prefecture-level cities in China and found that resource abundance is a “curse” to green economic growth. Cheng et al. [29] used the Malmquist–Luenberger index to measure green TFP at the provincial level in China and found that resource industry dependence negatively affects the green growth of the economy. The phenomenon mainly occurred through the extrusion of investment in innovation and human capital, hindering industrial development and reducing the quality of local systems.

The resource curse phenomenon occurs across the entire economic and social system, and its mechanism is affected by other external factors, and country’s openness to international markets was proved to be one of the essential factors [30,31]. In recent years, China’s economy has entered a new period. China has gradually lost its comparative advantage in labor due to the increase in labor prices. Owing to the global manufacturing shift to Southeast Asia, the Sino–US trade war has reached an unstable condition, and the traditional growth model of relying on exports to drive the economy has been severely challenged. Therefore, to investigate the existence of a resource curse in China, we could not ignore the moderating effect of the trade environment. Arezki and Ploeg [32] proved that natural resource endowments are negatively correlated with economic growth, but increasing trade openness can reduce this negative effect. Dong and Yan [33] used China’s provincial panel data from 1997 to 2012 as a sample, and found that the level of expansion has a threshold effect on the resource curse phenomenon; the level of expansion can effectively improve the relationship between resource endowment and economic growth. When the level of expansion is higher than the threshold, the abundance of resources does not hinder economic growth. These studies have identified the moderating effect of trade level on the resource curse, but they have not specifically analyzed the route through which trade level changes the relationship between resource endowment and green growth. Therefore, in this study, we decomposed green TFP into the effects of energy conservation and environmental improvement to analyze the specific route through which the import and export threshold effects change the resource curse mechanism.

## 3. Materials and Methods

### 3.1. Measurement and Decomposition of Green Total Factor Productivity

#### 3.1.1. Super-Efficiency Data Envelopment Analysis (DEA) Model

In this study, green growth is characterized by green total factor productivity (TFP). In order to calculate green TFP of each province in China, we first need to measure the level of inefficiency relevant to energy and the environment. Data envelopment analysis (DEA) is a commonly used relative efficiency evaluation model. Charnes et al. [34] proposed the first DEA model, termed the CCR-DEA model, which is an efficiency measurement method based on the assumption of constant returns to scale. Banker et al. [35] modified the CCR-DEA model and proposed a BCC-DEA model based on the assumption of variable returns to scale. When such traditional DEA models are used to evaluate the efficiency of decision-making units, multiple decision-making units may be at the forefront of input and output simultaneously, and the traditional DEA models cannot efficiently rank multiple effective units. To overcome this shortcoming, Andersen et al. [36] proposed a super-efficiency DEA model, which is based on the radial directional distance function for planning and solving, requiring input or output to approach the frontier with the same ratio. The nonradial directional distance function considers the relaxation of variables, allowing input and output to shrink and expand at different proportions. Therefore, this study improved upon the model proposed by Andersen et al. [36] and used the super-efficiency DEA model based on the nonradial directional distance function to measure the green TFP.

Equation (1) represents a super-efficiency DEA model based on a nonradial directional distance function, considering the efficiency evaluation of the *i*th province in year *t* as an example.

$$D_{i}^{t}\left(k^{t},l^{t},y^{t},e^{t},u_{t};g^{t}\right)=\underset{\lambda ,\beta_{i,e\;}^{t},\;\beta_{i,u,j\;}^{t},s_{i,k\;}^{t},s_{i,l}^{t}}{max\;:\beta_{i}^{t}}=\omega_{e}\beta_{i,e}^{t}+\overset{3}{\underset{j=1}{\sum }}\omega_{u,j}\beta_{i,u,j}^{t}+\varepsilon_{k}s_{i,k}^{t}+\varepsilon_{l}s_{i,l}^{t}$$

(1) $$s.t.\left\{\begin{array}{c} \sum_{n=1,n\neq i}^{N}\lambda_{n}^{t}\times k_{n}^{t}+s_{i,k}^{t}\;\leq k_{i}^{t}\\ \sum_{n=1,n\neq i}^{N}\lambda_{n}^{t}\times l_{n}^{t}+s_{i,l}^{t}\;\leq l_{i}^{t}\\ \sum_{n=1,n\neq i}^{N}\lambda_{n}^{t}\times y_{n}^{t}\;\geq y_{i}^{t}\\ \sum_{n=1,n\neq i}^{N}\lambda_{n}^{t}\times e_{n}^{t}\leq \left(1-\beta_{i,e}^{t}\right)e_{i}^{t}\\ \sum_{n=1,n\neq i}^{N}\lambda_{n}^{t}\times u_{n,j}^{t}\leq \left(1-\beta_{i,u,j}^{t}\right)u_{i,j}^{t}\;j=1,2,3\\ \beta_{i,e}^{t},\beta_{i,u,j}^{t}\leq 1\\ \lambda_{n}^{t}\geq 0 \end{array}\right.$$

The main difference between the super-efficient and the traditional DEA models is that in the super-efficient DEA model, the efficiency of the *i*th province must be excluded from the set of decision-making units, that is, the *i*th province does not contribute to the process of building the frontier. In Equation (1), $D_{i}^{t}$ represents the directional distance function of the *i*th province in year *t*, *N* represents the total number of provinces, and $\lambda_{n}^{t}\geq 0$ represents that the model satisfies the assumption of constant returns to scale; $k_{n}^{t}$, $l_{n}^{t}$, $y_{n}^{t}$, $e_{n}^{t}$, and $u_{n,j}^{t}$ denote the capital input, labor input, desired output, energy input, and undesired output of the *n*th $\left(n=1,2\ldots ,N;n\neq i\right)$ province in year *t*, respectively, in which $j\left(j=1,2,3\right)$ indicates that there are three types of undesired outputs. The term *g^t^* is the direction vector, indicating the directions of input and output optimization; in this study, $g^{t}=\left(0,0,0,-e_{i}^{t},-u_{i,1}^{t},-u_{i,2}^{t},-u_{i,3}^{t}\right)$. $s_{i,k}^{t}$ and $s_{i,l}^{t}$ denote the slack variables of capital input and labor input, respectively; $\beta_{i,e}^{t}$ and $\beta_{i,u,j}^{t}$ denote the ratio of energy input and undesired output that need to be reduced to reach the production frontier level in the *i*th province. A positive value of $\beta_{i,e}^{t}$ or $\beta_{i,u,j}^{t}$ indicates the inefficiency level of energy input and undesired output, while a negative value indicates the super-efficiency level. Here, it is not required that $\beta_{i,e}^{t}$ and $\beta_{i,u,j}^{t}$ be equal, or that the energy input and undesired output change in the same proportion; $\beta_{i}^{t}$ denotes the level of inefficiency in the *i*th province in the *t*th year, which equals the weighted average of the above four inefficiency values as well as $\varepsilon_{k}s_{i,k}^{t}$ and $\varepsilon_{l}s_{i,l}^{t}$. The optimized objective function was used to maximize the $\beta_{i}^{t}$. The weights of $\beta_{i,e}^{t}$ and $\beta_{i,u,j}^{t}$ are $\omega_{e}$ and $\omega_{u,j}$ ($\omega_{e}+\overset{3}{\underset{j=1}{\sum }}\omega_{u,j}=1$), respectively. Because the efficiency level is evaluated from the perspectives of energy conservation and environmental improvement, we assigned the weight $\omega_{e}$ of the energy inefficiency level $\beta_{i,e}^{t}$ to 1/2, and the weights $\omega_{u,j}$ (*j* = 1,2,3) of the three undesired output inefficiencies were 1/6. Variables $\varepsilon_{k}$ and $\varepsilon_{l}$ are the non-Archimedean infinitesimal quantities. In the objective function, the inefficiency level of capital and labor inputs are denoted by $\varepsilon_{k}s_{i,k}^{t}$ and $\varepsilon_{l}s_{i,l}^{t}$, respectively, which are the products of a finite constant and a non-Archimedean infinitesimal. Their values remain infinitesimal, and these do not have a significant effect on the objective function $\beta_{i}^{t}$.

#### 3.1.2. Luenberger Green Total Factor Productivity Index and Decomposition

Equation (1) measures the inefficiency level using the nonradial directional distance function. Because of the additive form of the nonradial directional distance function, green TFP can be constructed by the results of inefficiency through the Luenberger productivity index [37]. Green TFP refers to the level of change in green efficiency in the current period, based on the previous period. A green TFP greater than zero indicates an increase in green efficiency, and a value less than zero indicates a decline in green efficiency. We assumed that the previous period is recorded as period 0, and the current period is recorded as period 1. The Luenberger green TFP ($L_{0,i}^{1}$) of the *i*th province follows.

(2) $$L_{0,i}^{1}=\frac{1}{2}\times \left[D_{i}^{1}\left(k^{0},l^{0},y^{0},e^{0},u^{0};g^{0}\right)-D_{i}^{1}\left(k^{1},l^{1},y^{1},e^{1},u^{1};g^{1}\right)+D_{i}^{0}\left(k^{0},l^{0},y^{0},e^{0},u^{0};g^{0}\right)-D_{i}^{0}\left(k^{1},l^{1},y^{1},e^{1},u^{1};g^{1}\right)\right]$$

Luenberger green TFP comprises four nonradial directional distance functions, of which the same-phase directional distance functions $D_{i}^{0}\left(k^{0},l^{0},y^{0},e^{0},u^{0};g^{0}\right)$ and $D_{i}^{1}\left(k^{1},l^{1},y^{1},e^{1},u^{1};g^{1}\right)$ are shown in Equations (3) and (4), respectively.

$$D_{i}^{0}\left(k^{0},l^{0},y^{0},e^{0},u^{0};g^{0}\right)=max\;:\;\beta_{i}^{0,0}=\omega_{e}\beta_{i,e}^{0,0}+\overset{3}{\underset{j=1}{\sum }}\omega_{u,j}\beta_{i,u,j}^{0,0}+\varepsilon_{k}s_{i,k}^{0,0}+\varepsilon_{l}s_{i,l}^{0,0}$$

(3) $$s.t.\left\{\begin{array}{c} \sum_{n=1,n\neq i}^{N}\lambda_{n}^{0,0}\times k_{n}^{0}+s_{i,k}^{0,0}\;\leq k_{i}^{0}\\ \sum_{n=1,n\neq i}^{N}\lambda_{n}^{0,0}\times l_{n}^{0}+s_{i,l}^{0,0}\;\leq l_{i}^{0}\\ \sum_{n=1,n\neq i}^{N}\lambda_{n}^{0,0}\times y_{n}^{0}\;\geq y_{i}^{0}\\ \sum_{n=1,n\neq i}^{N}\lambda_{n}^{0,0}\times e_{n}^{0}\leq \left(1-\beta_{i,e}^{0,0}\right)e_{i}^{0}\\ \sum_{n=1,n\neq i}^{N}\lambda_{n}^{0,0}\times u_{n,j}^{0}\leq \left(1-\beta_{i,u,j}^{0,0}\right)u_{i,j}^{0}\;j=1,2,3\\ \beta_{i,e}^{0,0},\beta_{i,u,j}^{0,0}\leq 1\\ \lambda_{n}^{0,0}\geq 0 \end{array}\right.$$

$$D_{i}^{1}\left(k^{1},l^{1},y^{1},e^{1},u^{1};g^{1}\right)=max\;:\;\beta_{i}^{1,1}=\omega_{e}\beta_{i,e}^{1,1}+\overset{3}{\underset{j=1}{\sum }}\omega_{u,j}\beta_{i,u,j}^{1,1}+\varepsilon_{k}s_{i,k}^{1,1}+\varepsilon_{l}s_{i,l}^{1,1}$$

(4) $$s.t.\left\{\begin{array}{c} \sum_{n=1,n\neq i}^{N}\lambda_{n}^{1,1}\times k_{n}^{1}+s_{i,k}^{1,1}\;\leq k_{i}^{1}\\ \sum_{n=1,n\neq i}^{N}\lambda_{n}^{1,1}\times l_{n}^{1}+s_{i,l}^{1,1}\;\leq l_{i}^{1}\\ \sum_{n=1,n\neq i}^{N}\lambda_{n}^{1,1}\times y_{n}^{1}\;\geq y_{i}^{1}\\ \sum_{n=1,n\neq i}^{N}\lambda_{n}^{1,1}\times e_{n}^{1}\leq \left(1-\beta_{i,e}^{1,1}\right)e_{i}^{1}\\ \sum_{n=1,n\neq i}^{N}\lambda_{n}^{1,1}\times u_{n,j}^{1}\leq \left(1-\beta_{i,u,j}^{1,1}\right)u_{i,j}^{1}\;j=1,2,3\\ \beta_{i,e}^{1,1},\beta_{i,u,j}^{1,1}\leq 1\\ \lambda_{n}^{1,1}\geq 0 \end{array}\right.$$

In the interperiod program, because the set of decision-making units and the decision-making unit being evaluated were not from the same period of data, the interperiod data of the *i*th province were not excluded from the set of decision-making units. The super-efficiency DEA model can not only sort multiple effective decision-making units, but also solve the problem of unsolvable intertemporal planning. The intertemporal directional distance functions $D_{i}^{0}\left(k^{1},l^{1},y^{1},e^{1},u^{1};g^{1}\right)$ and $D_{i}^{1}\left(k^{0},l^{0},y^{0},e^{0},u^{0};g^{0}\right)$ are shown in Equations (5) and (6), respectively.

$$D_{i}^{0}\left(k^{1},l^{1},y^{1},e^{1},u^{1};g^{1}\right)=max\;:\;\beta_{i}^{0,1}=\omega_{e}\beta_{i,e}^{0,1}+\overset{3}{\underset{j=1}{\sum }}\omega_{u,j}\beta_{i,u,j}^{0,1}+\varepsilon_{k}s_{i,k}^{0,1}+\varepsilon_{l}s_{i,l}^{0,1}$$

(5) $$s.t.\left\{\begin{array}{c} \sum_{n=1}^{N}\lambda_{n}^{0,1}\times k_{n}^{0}+s_{i,k}^{0,1}\;\leq k_{i}^{1}\\ \sum_{n=1}^{N}\lambda_{n}^{0,1}\times l_{n}^{0}+s_{i,l}^{0,1}\;\leq l_{i}^{1}\\ \sum_{n=1}^{N}\lambda_{n}^{0,1}\times y_{n}^{0}\;\geq y_{i}^{1}\\ \sum_{n=1}^{N}\lambda_{n}^{0,1}\times e_{n}^{0}\leq \left(1-\beta_{i,e}^{0,1}\right)e_{i}^{1}\\ \sum_{n=1}^{N}\lambda_{n}^{0,1}\times u_{n,j}^{0}\leq \left(1-\beta_{i,u,j}^{0,1}\right)u_{i,j}^{1}\;j=1,2,3\\ \beta_{i,e}^{0,1},\beta_{i,u,j}^{0,1}\leq 1\\ \lambda_{n}^{0,1}\geq 0 \end{array}\right.$$

$$D_{i}^{1}\left(k^{0},l^{0},y^{0},e^{0},u^{0};g^{0}\right)=max\;:\;\beta_{i}^{1,0}=\omega_{e}\beta_{i,e}^{1,0}+\overset{3}{\underset{j=1}{\sum }}\omega_{u,j}\beta_{i,u,j}^{1,0}+\varepsilon_{k}s_{i,k}^{1,0}+\varepsilon_{l}s_{i,l}^{1,0}$$

(6) $$s.t.\left\{\begin{array}{c} \sum_{n=1}^{N}\lambda_{n}^{1,0}\times k_{n}^{1}+s_{i,k}^{1,0}\;\leq k_{i}^{0}\\ \sum_{n=1}^{N}\lambda_{n}^{1,0}\times l_{n}^{1}+s_{i,l}^{1,0}\;\leq l_{i}^{0}\\ \sum_{n=1}^{N}\lambda_{n}^{1,0}\times y_{n}^{1}\;\geq y_{i}^{0}\\ \sum_{n=1}^{N}\lambda_{n}^{1,0}\times e_{n}^{1}\leq \left(1-\beta_{i,e}^{1,0}\right)e_{i}^{0}\\ \sum_{n=1}^{N}\lambda_{n}^{1,0}\times u_{n,j}^{1}\leq \left(1-\beta_{i,u,j}^{1,0}\right)u_{i,j}^{0}\;j=1,2,3\\ \beta_{i,e}^{1,0},\beta_{i,u,j}^{1,0}\leq 1\\ \lambda_{n}^{1,0}\geq 0 \end{array}\right.$$

Mahlberg et al. [37] and Chang et al. [38] proposed that the Luenberger productivity index based on the nonradial directional distance function can be decomposed into the sum of the productivity of each factor. The Luenberger green TFP in this study can be decomposed into energy conservation and environmental improvement effects (because $\varepsilon_{k}s_{i,k}^{t}$ and $\varepsilon_{l}s_{i,l}^{t}$ are infinitesimal, they can be ignored). $L_{0,i}^{1}$ represents the green TFP; $L_{0,i,e}^{1}$ and $L_{0,i,u}^{1}$ represent the efficiency changes of energy input and undesired output, that is, the energy conservation and environmental improvement effects, respectively.

(7) $$L_{0,i,e}^{1}=\frac{1}{2}\times \left[\left(\beta_{i,e}^{1,0}-\beta_{i,e}^{1,1}\right)+\left(\beta_{i,e}^{0,0}-\beta_{i,e}^{0,1}\right)\right]$$

(8) $$L_{0,i,u}^{1}=\frac{1}{2}\times \left[\left(\overset{3}{\underset{j=1}{\sum }}\frac{1}{3}\beta_{i,u,j}^{1,0}-\overset{3}{\underset{j=1}{\sum }}\frac{1}{3}\beta_{i,u}^{1,1}\right)+\left(\overset{3}{\underset{j=1}{\sum }}\frac{1}{3}\beta_{i,u,j}^{0,0}-\overset{3}{\underset{j=1}{\sum }}\frac{1}{3}\beta_{i,u}^{0,1}\right)\right]$$

(9) $$L_{0,i}^{1}=\frac{1}{2}\times \left(L_{0,i,e}^{1}+L_{0,i,u}^{1}\right)$$

#### 3.1.3. Input–Output Data in the Measurement of Green TFP

This study utilized the input–output data of 30 provinces and regions in China for 2005 to 2017. Because of the unavailability of data, the study did not include Tibet, Hong Kong, Macao, and Taiwan among the 34 provinces and regions of China. Moreover, Beijing, Tianjin, Shanghai, and Chongqing were not excluded from the calculation of TFP; however, because the functional positioning of municipalities is different from that of provinces and autonomous regions, the data of these four municipalities were excluded when calculating the threshold variable using the panel model. The data included capital input (*k*), labor input (*l*), energy input (*e*), expected output (*y*), and undesired output (*u*), whose sources and processing methods are explained as follows.

Capital input (*k*): We estimated the annual capital stock based on the perpetual inventory method proposed by Zhang et al. [39]. The earlier the selected base year, the lower the effect that the error of the initial capital stock estimated during the base year has in subsequent years. Therefore, 1952 was selected as the base year for estimation. The fixed asset depreciation rate of all provinces and regions was uniformly set to 9.6%, the total fixed capital formation was used as the current investment amount, and the regional fixed asset investment price index was used to convert the fixed asset investment price index into a constant price with 2005 as the base year.

Labor input (*l*): If the number of the employed population is used to represent labor input, the differences due to different levels of education can be ignored. Therefore, labor input was selected as the product of the total employed population in the primary, secondary, and tertiary industries and the average years of education in the region.

Energy input (*e*): Energy input represents the total energy consumption by region published in the *China Energy Statistical Yearbook*.

Desirable output (*y*): Desirable output is the gross domestic product (GDP), with 2005 as the base period, and the GDP index was used to account for deflation.

Undesirable output (*u*): Undesirable outputs include total SO_2_ emissions, total wastewater emissions, and solid waste generation in each province.

The data were mainly obtained from the *China Statistical Yearbook*, *China Labor Statistics Yearbook*, *China Energy Statistics Yearbook*, statistical yearbooks of various provinces, and Wind Economic Database.

### 3.2. Methodology and Data

#### 3.2.1. Model Settings

First, the linear relationship between resource endowment (*re*) and green TFP (*tfp*) should be examined. Because this study adopts panel data, and regions’ individual fixed effects and time fixed effects need to be controlled in the regression process, a fixed-effect model was used to perform a basic regression analysis to test the existence and regional heterogeneity of the resource curse. Then, considering the existence of a nonlinear relationship between resource endowment (*re*) and green TFP (*tfp*) with certain variables as moderators and applying import and export trade levels as threshold variables, the panel threshold model was used to identify the changes in the mechanism of the impact of resource endowments on green growth before and after the threshold value.

Because green TFP can be decomposed into the energy conservation effect (*tfp_e*) and the environmental improvement effect (*tfp_u*), we also examined the influence of resource endowment (*re*) on the two effects in both models above to identify the route by which the resource curse phenomenon changes.

The fixed-effects model is shown in Equation (10):

(10) $$gg_{i,t}=\alpha_{0}+\alpha_{1}re_{i,t}+\alpha_{2}control_{i,t}+\mu_{i}+\lambda_{t}+\varepsilon_{i,t}$$

where *i* denotes the province and *t* denotes the year. The explanatory variable gg denotes the green growth effect, and the *tfp*, *tfp_e*, and *tfp_u* can be selected according to different study objectives. The core explanatory variable re is the resource endowment; *control* denotes the control variable, including imports, exports, environmental governance, research and development (R&D) investment, economic development level, industrial structure, urbanization level, and nationalization level; *α*_0_ is a constant term; *α*_1_ and *α*_2_ are the regression coefficients for the explanatory and control variables, respectively; $\mu_{i}$ is a fixed effect in a region that does not change with time; $\lambda_{t}$ denotes a fixed effect in time; and $\varepsilon_{i,t}$ is a random perturbation term.

As previously mentioned, import and export trade levels may play a threshold role in the resource curse hypothesis; however, it is difficult to determine the specific segmentation point. Therefore, the threshold regression model proposed by Hansen [40] was used for analysis. This model can accurately estimate the threshold value and perform a significant test of the threshold effect. The panel threshold model is shown in Equation (11):

(11) $$gg_{i,t}=\alpha_{0}+\alpha_{11}re_{i,t}\cdot I\left(q\leq \gamma \right)+\alpha_{12}re_{i,t}\cdot I\left(q>\gamma \right)+\alpha_{2}control_{i,t}+\mu_{i}+\varepsilon_{i,t}$$

where q is the threshold variable, which represents the level of import and export trade, respectively, and γ is a threshold value. $I\left(*\right)$ is an indicative function; if the expression within the parentheses is true, the value is one, and the opposite is zero. When the threshold variable is lower than the threshold value $\left(q\leq \gamma \right)$, *α*_11_ is the regression coefficient of the resource endowment, and when the threshold variable is above the threshold value $\left(q>\gamma \right)$, *α*_12_ is the regression coefficient of the resource endowment. The meanings of the interpreted, explanatory, and control variables in Equation (11) are the same as those in Equation (10).

Equation (11) is the expression of a single threshold model, and if the threshold effect test proves the occurrence of a double threshold or triple threshold, a corresponding multithreshold model should also be established. Considering the double threshold model as an example, the corresponding expression is shown in Equation (12):

(12) $$gg_{i,t}=\alpha_{0}+\alpha_{11}re_{i,t}\cdot I\left(q\leq \gamma_{1}\right)+\alpha_{12}re_{i,t}\cdot I\left(\gamma_{1}<q\leq \gamma_{2}\right)+\alpha_{13}re_{i,t}\cdot I(q>\gamma_{2})+\alpha_{2}control_{i,t}+\mu_{i}+\varepsilon_{i,t}$$

where *α*_11_, *α*_12_, and *α*_13_ are the regression coefficients of resource endowments under different threshold intervals, *q* is a threshold variable, and $\gamma_{1}$ and $\gamma_{2}$ are two different threshold values.

Stata 14.0 statistical analysis software was used to estimate the model.

#### 3.2.2. Data Sources

The definitions and descriptions of the variables involved in the model are presented in Table 1.

Explained variables, including green total factor productivity (*tfp*), energy conservation effect (*tfp_e*), and environmental improvement effect (*tfp_u*), are derived from the model and data in 3.1.

Explanatory variable is resource endowment (*re*). The number of employees in the mining industry was chosen to be the proxy variable of resource endowment, because it can reflect the dependence of a region’s economic development on resources and the abundance of resources [41]. The data are derived from the *China Labor Statistics Yearbook*. The impact of mineral resources on green growth was mainly considered in this study. Owing to the low-cost utilization of mineral resources, the regions rich in mineral resources lack the motivation for green production technology innovation, which may not be conducive to green growth. Although renewable energy is part of natural resource endowment, the development and utilization of renewable energy requires a high level of technology, which rarely causes a “curse” to the economy, and it accounts for a small proportion in energy consumption; thus, this study does not consider such resources.

The calculation methods of control variables, including imports, exports, environmental governance, research and development (R&D) investment, economic development level, industrial structure, urbanization level, and nationalization level, are presented in Table 1. Except for the stock of R&D capital (*rd*), the data used to calculate other control variables are directly derived from the *China Statistical Yearbook*, *China Labor Statistics Yearbook*, *China Population and Employment Statistical Yearbook*, and statistical yearbooks of various provinces. Because the government has not released statistics on the stock of R&D capital, we used the perpetual inventory method to estimate the calculation equation as follows:

(13) $$S_{i,t}=\left(1-\delta \right)S_{i,t-1}+RD_{i,t}$$

where $S_{i,t}$ and $S_{i,t-1}$ are the R&D capital stocks of province *i* in year *t* and *t* − 1, respectively; and $RD_{i,t}$ is the internal R&D expenditure of province *i* in year *t*. The term δ is the depreciation rate, which is consistent with the previous estimate of capital stock, and is also set to 9.6%. Considering 2000 as the initial year, the calculation method for capital stock in 2000 follows:

(14) $$S_{i,2000}=RD_{i,2000}/\left(\delta +g\right)$$

where $S_{i,2000}$ is the R&D capital stock of province *i* in 2000, $RD_{i,2000}$ is the internal expenditure of R&D expenditure in province *i* in 2000, δ is the depreciation rate (9.6%), and g represents the average growth rate of internal R&D expenditure from 2000 to 2017.

## 4. Results and Discussion

### 4.1. Green Total Factor Productivity Levels in Each Province and Region

This study used the Linprog function in MATLAB to calculate the green TFP. Equation (9) shows that green TFP can be decomposed into energy conservation and environmental improvement effects. Table 2 shows the average values (2005–2017) of the three indicators—*tfp*, *tfp_e*, and *tfp_u*—in each province. From the national average result, the green TFP is 0.062%, of which the negative energy conservation effect leads to an average annual decline of 0.409% in green TFP, but the environmental improvement effect contributes 0.472% of the increase in green TFP. According to the specific conditions of each province, the green TFP of Beijing (7.783%) and Shanghai (2.510%) were significantly higher than those of other provinces, while those of Heilongjiang, Hainan, and Xinjiang were all less than −1.000%. However, the growth effect was negative. For most provinces, environmental improvement was the main reason for the increase in green TFP, while the decline in energy use efficiency hindered green growth. However, the energy conservation effects of Beijing, Shanxi, and Jilin were positive, indicating that the energy use efficiencies of these three provinces have increased, which in turn increased the green TFP. The environmental improvement effects of Heilongjiang, Qinghai, and Ningxia were negative. For these provinces, the deterioration of environmental efficiency was the main reason for the decline in TFP.

### 4.2. Descriptive Statistics of the Variables in Fixed-Effects Model

To understand the variables more intuitively, Table 3 lists the descriptive statistics for each. Because the functional positioning of municipalities is different from that of provinces and autonomous regions, the sample data of Beijing, Tianjin, Shanghai, and Chongqing were excluded from the follow-up empirical research.

### 4.3. Analysis of the Existence and Regional Differences of the Resource Curse

First, regardless of the influence of threshold variables on the mechanism of the resource curse, a fixed-effect model (Equation (10)) was used to examine the linear relationship between resource endowment and green growth. Model 1, Model 2, and Model 3 describe the impact of resource endowment (*re*) on *tfp*, *tfp_e*, and *tfp_u* under the full sample, respectively, and the existence of the resource curse hypothesis was tested from these three aspects. Models 4, 5, and 6 are the estimation models of the impact of resource endowments in the eastern, central, and western regions on *tfp*, respectively, testing the regional heterogeneity of the resource curse. The eastern region includes Hebei and Liaoning, Jiangsu, Zhejiang, Fujian, Shandong, Guangdong, and Hainan; the central region includes Shanxi, Jilin, Heilongjiang, Anhui, Jiangxi, Henan, Hubei, and Hunan; and the western region includes Inner Mongolia, Guangxi, Sichuan, Guizhou, Yunnan, Shaanxi, Gansu, Qinghai, Ningxia, and Xinjiang. Table 4 presents the regression results of the fixed-effects model.

According to the regression results of the full sample in Table 4, a significant negative correlation exists between resource endowment and green TFP, energy conservation effect, and environmental improvement effect. From a national perspective, the resource curse hypothesis was found to be valid, and abundant natural resources hinder energy conservation and environmental improvement, while negatively affecting green growth through the effects of energy and the environment. However, regional heterogeneity was observed in the phenomenon of the resource curse. The regression results in the eastern region showed that resource endowment had a significant positive impact on green TFP. In the eastern region, abundant natural resources are conducive to increasing the level of green economic growth. The results for the central region are similar to those of the full sample. The central region also exhibited the resource curse phenomenon, but the negative correlation between resource endowments and green TFP in the central region was less than that in the full sample. Moreover, the severity was lower than that at the national level. The resource endowment in the western region did not significantly affect the green TFP; thus, the resource curse hypothesis was not true for this region. In summary, resource endowment is a “blessing” for the eastern region and a “curse” for the central region, but they do not affect the green growth effect in the western region.

Regression results from other control variables showed that imports would not have a significant impact on green growth at the national and regional levels, but exports would negatively affect green TFP in the western region. Environmental governance would have a significant negative impact on green TFP, energy conservation effect, and environmental improvement effect, and the increase in environmental governance is not conducive to green economic growth. Environmental governance can reflect the strength of local environmental regulations and environmental access standards to a certain extent. If the environmental regulations of a region are too strict or environmental access standards are too high, some polluting enterprises cannot enter the local market, resulting in damage to the output structure. Therefore, environmental regulation in China has not yet demonstrated an innovative compensation effect according to the Porter hypothesis [42].

The level of urbanization had a significant positive impact on the TFP and energy conservation effects. Increasing the level of urbanization helps achieve green economic growth and energy conservation. The industrial structure was positively related to the effect of environmental improvement; thus, the higher the proportion of the secondary industry, the greater the degree of improvement in environmental quality. However, this is contrary to people’s traditional cognition, but it can be explained reasonably from two perspectives. ① Even if the country has been emphasizing the adjustment of industrial structure, it cannot ignore the role of the secondary industry as a pillar of China’s economic development. Increasing the proportion of secondary industries can increase the level of green growth by increasing output. ② Environmental quality in areas with heavy industries is generally low, leading to greater opportunities for environmental improvement.

### 4.4. Transformation of the Resource Curse Mechanism and Analysis of the Mechanism under the Import Level Threshold

Import trade is not only a supplementary means to improve the structure of domestic consumer goods supply, but it is also an important method to determine technology spillovers. However, excessive dependence on imported products reduces domestic manufacturing. Therefore, import trade has two opposite effects on the economy: the technology spillover and the product crowding-out effects. Import trade not only affects the domestic product structure but may also indirectly affect the energy structure and environmental quality. Therefore, “import” was used as a threshold variable to further analyze the nonlinear relationship between resource endowments and green growth. In the following, Models 7, 8, and 9 used imports as the threshold variable. The explained variables of the three models are *tfp*, *tfp_e*, and *tfp_u*. Among them, Model 7 was used to identify the mechanism change of the resource curse under different import levels, and Models 8 and 9 were used to identify the route through which the import trade promotes the mechanism change of the resource curse.

Table 5 shows the analysis results for the import threshold effect. Both Models 7 and 8 had significant single threshold effects, but no double threshold effect was observed. Therefore, for both Models 7 and 8, a single threshold model was adopted with import as the threshold variable (Equation (11)). However, the single threshold effect of Model 9 did not pass the 10% significance level test; therefore, there was no threshold effect, indicating that there was no nonlinear relationship between resource endowment and environmental improvement effects when “import” was the threshold variable. Therefore, Model 9 became equivalent to Model 3 (the fixed-effects model), and it is not discussed further.

The F-statistic and the critical value of P were simulated by repeated sampling (500 times) using the bootstrap method.

**Figure 1:**
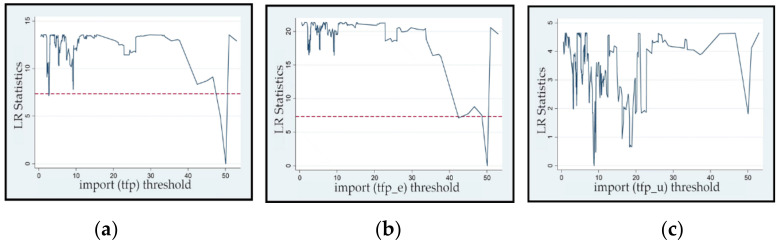
Likelihood ratio (LR) function graph of the import threshold variables: (**a**) green TFP (*tfp*), (**b**) energy conservation effect (*tfp_e*), and (**c**) environmental improvement effect (*tfp_u*).

Table 6 shows the estimated value and confidence interval of the import threshold in Models 7 and 8. Because Model 9 did not have a threshold effect, there is no corresponding estimated value or confidence interval. Figure 1a–c shows the images of the likelihood ratio functions of the import trade threshold variables in the three models. The threshold values of Models 7 and 8 were the same (50.110), which implies that the impact of resource endowment on green TFP and energy conservation effects both undergo a mechanism change at approximately 50.110; that is, when the total imports accounted for more than 50.110% of GDP, the resource curse changed its mechanism. However, the impact of resource endowments on the mechanism of environmental improvement effects did not change under different import levels. In summary, the mechanism of resource curse changes when the import level is at different ranges; however, imports can only change the impact of resource endowment on green growth through the route of energy conservation, and the behavior of resource curse on the mechanism of environmental improvement has not changed.

Table 7 shows the regression results for Models 7 and 8. The regression results of Model 7 revealed that when the ratio of total imports to GDP was less than 50.110%, the regression coefficient of resource endowment to green TFP was −0.084, and it passed the 1% level of significance test; however, when the import level exceeded the threshold value, the regression coefficient of resource endowment was −0.636, and the negative impact of resource endowment on green TFP increased significantly. In the regression results of Model 8, when the import level did not exceed the threshold value, that is, when the ratio of total imports to GDP was less than 50.110%, no significant correlation was found between resource endowment and the energy conservation effect; however, when the ratio exceeded 50.110%, resource endowment had a negative impact on the energy conservation effect. In summary, an increase in the level of import trade intensifies the adverse impact of resource endowment on green growth and promotes the deterioration of the resource curse. When the import level exceeded the threshold value, the resource curse phenomenon occurred along the energy route. This shows that import trade hinders energy conservation, which in turn leads to the deterioration of the resource curse, while imports do not change the relationship between resources and development through the route of environmental improvement. From the perspective of the import commodity structure in China, the proportion of primary product imports in 2015, 2016, and 2017 accounted for 28.11%, 27.78%, and 31.44%, respectively. Raw materials and fossil fuels are the main imported primary products, in which raw oil imports account for approximately 8% of the total import value (the import ratio of primary products and raw oil is manually calculated based on data from the *China Statistical Yearbook* [43]). The high proportion of imports of primary products leads to weaker technology spillover effects of import trade, hindering the increase in green TFP in China through import trade. The higher the import trade level of a province, the higher the dependence of the province’s economic growth on the resources of other countries. International trade has solved the scarcity of resources in the region to a certain extent, but the cost of importing raw materials and energy also has a crowding-out effect on R&D investment in production technology. Owing to the large uncertainty and positive externalities in green technological innovation, when the supply of raw materials and energy in the international market is sufficient, most production entities attempt to solve the problem of scarcity of production materials through imports rather than through technological innovation to save more energy. Therefore, imports intensify the adverse effects of the resource curse by hindering technological innovation, and the high proportion of energy imports leads to imports that can impede energy conservation to promote the mechanism of the resource curse. Table 7 also shows that the regression coefficient of imports in Model 8 is 0.095, and it is significant at the 10% level. This shows that even though import trade had a direct positive effect on TFP, the absolute value of this positive effect was lower than the absolute value of the negative effect from resource curse (the effect of resource endowment on TFP) when the import trade level exceeded the threshold value. To effectively reflect the positive role of import trade on TFP and avoid the occurrence of the resource curse, the level of import trade should be controlled below the threshold value.

### 4.5. Transformation of the Resource Curse Mechanism and Analysis of Mechanism under the Export Level Threshold

Export trade is an important method for a country to participate in international trade and exert its comparative advantages. Export trade can not only directly affect green growth, but also indirectly by affecting the relationship between resources and green growth. We used export trade as the threshold variable to find the different impacts of resource endowment on green TFP under different export levels, and the specific mechanism for this difference. Export trade was used as the threshold variable in Models 10, 11, and 12. The explained variables of the three models are *tfp*, *tfp_e*, and *tfp_u*. The role of Model 10 was to identify the change of the resource curse mechanism under different export levels, and the roles of Models 11 and 12 were to identify the route through which the export leads to the mechanism change of the resource curse.

Table 8 presents the analysis results for the export threshold effect. Models 10 and 12 had a significant single threshold effect, and both passed the 10% significance level test. In contrast, the double threshold effect of the two models did not pass the significance test; therefore, a single threshold model with export trade as the threshold variable should be used (Equation (11)). However, Model 11 did not pass the single threshold test, indicating that there is no nonlinear relationship between resource endowment and the energy conservation effect with export trade as the threshold variable. Therefore, Model 11 became equivalent to Model 2 (the fixed-effects model), and it is not discussed further.

The F-statistic and the critical value of *p* were simulated by repeated sampling (500 times) using the bootstrap method.

**Figure 2:**
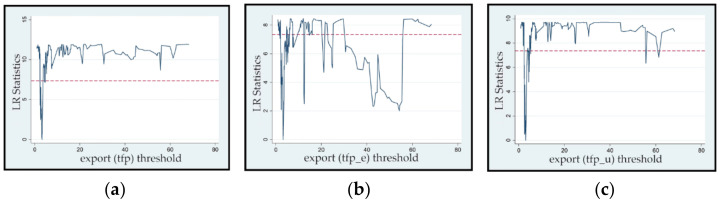
LR function graph of export threshold variables: (**a**) *tfp*, (**b**) *tfp_e*, and (**c**) *tfp_u*.

Table 9 shows the estimated value and confidence interval of the export threshold in Models 10 and 12. Because Model 11 does not have a threshold effect, there is no corresponding estimated value or confidence interval. Figure 2a–c shows the likelihood ratio (LR) functions of the exit threshold variables in the three models. The threshold values of Models 10 and 12 were 3.232 and 3.076, respectively. When the ratio of the total export trade to GDP exceeded 3.076%, the effect of resource endowment on environmental improvement changed, and when the ratio exceeded 3.232%, the effect of resource endowment on green TFP changed. Because there is no threshold effect in Model 11, the impact of resource endowments on energy conservation effects did not change at different export levels. In summary, when the export level is at different ranges, the mechanism of the resource curse changes. However, exports can only change the impact of resource endowment on green growth by hindering the mechanism of environmental improvement, and the resource curse changes, but exports can only change the impact of resource endowment on green growth by hindering the mechanism of environmental improvement.

Table 10 shows the regression results of Models 10 and 12. From the results of Model 10, when the export level did not exceed the threshold value, that is, when the ratio of total exports to GDP was lower than 3.232%, a significant negative correlation existed between resource endowment and green TFP, with a regression coefficient of −0.184. However, when the export level exceeds the threshold of 3.232%, the regression coefficient of resource endowment was −0.094, and passed the 1% significance level test. Moreover, when the export level increased to the threshold value, the negative impact of resource endowment on green TFP was weakened. The regression results of Model 12 showed that when the ratio of total exports to GDP was lower than 3.076%, the correlation coefficient between resource endowment and environmental improvement effect was −0.233; however, when the export level exceeded the threshold value, the regression coefficient of resource endowment was −0.136. Furthermore, with the increase in export level, the negative effect of resource endowment on the environmental improvement was also be weakened. In summary, export trade can reduce the adverse impact of resource endowment on green growth and alleviate the severity of the resource curse phenomenon. However, export trade can only change the relationship between resources and development through the route of environmental improvement, but not that of energy conservation. The structure of China’s export commodities in 2015, 2016, and 2017 revealed that the exports of industrial finished products accounted for 95.43%, 94.99%, and 94.80%, respectively, among which the export of machinery and transportation equipment accounted for a large proportion (the export ratio of industrial finished products is manually calculated based on data from the *China Statistical Yearbook* [34]). The continuous increase in the proportion of exports of heavy industrial products, such as machinery and equipment, indicates that the technological level of China’s export commodities is constantly improving. Environmental barriers and international market demand in export trade have caused Chinese companies to undergo technological innovation. China relies on industrial products to obtain export trade income, and the commodity structure of resource-dependent provinces is mostly based on raw materials and fossil fuels; hence, the resource-dependent provinces in China do not have evident trade advantages. However, owing to the large demand for such commodities in the domestic market, resource-based provinces and regions can obtain a comfortable living space even if they only serve the domestic market without export trade. The geographical distribution of China’s natural resources is uneven; the central and western regions are the areas with resource advantages, while the eastern regions rely on convenient transportation and trade conditions to ensure technological advantages. The separation of the resource and technological advantages has also led to differences in the division of labor between provinces and regions. The high level of export trade in the eastern region has intensified the demand for raw materials such as energy, which also increases the exploitation of natural resources by the central and western regions, deepens the dependence of the central and western regions on resources, and reduces the possibility of technological innovation in resource-dependent provinces. Unlike international trade, domestic trade cannot bring incentives for green technology innovation. Based on this analysis, the higher the export trade level of a province, the higher the industrial technology level of the region, and the less dependent the region is on resource endowments for economic development and green growth. The provinces with lower export levels mostly exhibited comparative advantages in terms of resources and lacked motivation for green technological innovation. Therefore, when the export level was lower than the threshold value, resource endowment had a severely negative impact on green growth. With the increase in the level of export trade, restrictions on environmental barriers have also continued to increase. High-exporting provinces give more attention to reducing the negative environmental externalities of the production process, while environmental barriers have less impact on provinces with low export levels; therefore, export trade can improve the resource curse phenomenon through the environmental improvement route. Table 10 also shows that even though the regression coefficient of exports is not significant, a negative correlation exists between exports and green TFP and environmental improvement effects. Therefore, the relationship between export trade and green growth should be adequately considered, and the Chinese government should allow export trade to play its role in improving the resource curse and adopt appropriate measures to eliminate its hindrance to green growth.

### 4.6. Limitations and Future Research

Although the study provided a useful conclusion concerning resource curse, the study still has some limitations. (1) Because of the availability of data, we choose the number of employees in the mining industry as the proxy variable of resource endowment, which may not fully represent the output scale of the mining industry. (2) In this study, green growth is characterized by green TFP. Although TFP has been widely used as an explained variable in the regression model [44], true TFP is unobserved and DEA estimates of productivity have their own limitations. (3) This study focuses on the threshold effect of trade level on the resource curse; thus, whether there is a relationship between green growth and the quadratic term of resources is not discussed.

Based on the results of the above analysis, we recommend the following future actions for Chinese government: (1) Because the resource curse phenomenon exists in China, the government should increase its efforts to promote economic green transformation and reduce the dependence of economic growth on natural resources. Because of the regional heterogeneity in the resource curse, the “one size fits all” approach should be avoided when implementing policies and regulations. For the eastern region, a policy promoting resource development should be implemented, while for the central region, a policy restricting resource development should be implemented. Although the mechanism of resource endowment in western China is not evident, the government should handle resource development activities cautiously and attempt to optimize the input factor structure from the supply side of resources. (2) Although increasing import trade level intensifies the resource curse, import trade also has a positive effect on environmental improvement. Therefore, the import trade level should be controlled below the threshold value and it must be ensured that the environmental dividend generated by import trade is fully utilized. (3) Although export trade could reduce the negative impact of the resource curse, its hindrance to green growth cannot be ignored. Hence, a reasonable level of export trade must be conducted in combination with economic development goals to alleviate resource dependence and mitigate its crowding-out effect on the output.

## 5. Conclusions

According to the resource curse hypothesis, abundant natural resources would become an obstacle to economic growth. Therefore, based on the concept of the resource curse phenomenon, this study attempts to interpret the relationship between resources and development from the perspective of green growth. However, the concept of development in this study is not limited to economic growth, but it evaluates the quality of economic development from multiple dimensions, such as energy conservation, environmental improvement, and economic growth. We re-examined the impact of resource endowments on green growth under the theoretical framework of the resource curse, and the level of green growth was indicated by the green TFP. Because there may be a nonlinear relationship between resource endowments and green growth with certain variables as moderators, the impact of import and export threshold effects on the transformation of the resource curse mechanism was further investigated, and the transformation route for the resource curse mechanism was identified from the perspectives of energy conservation and environmental improvement.

We provided evidence to support the resource curse hypothesis using a unique dataset of 26 provinces in China for 2005 to 2017 and applying them to a fixed-effects and a panel threshold model. The following are the analysis results. (1) The resource curse hypothesis was valid nationwide, and a significant negative correlation existed between resource endowments and green TFP, energy conservation effects, and environmental improvement effects. Resource endowments negatively affect green growth by hindering both energy conservation and environmental improvement. The phenomenon of resource curse exhibited regional heterogeneity; resource endowment was found to be a “blessing” for the eastern region, a “curse” for the central region, and did not affect the western region. (2) Import trade increased the adverse impact of resource endowment on green growth and promoted the deterioration of the resource curse situation. When the import level exceeded the threshold value, the resource curse phenomenon changed along the energy route. Import trade deteriorated the resource curse by impeding energy conservation, but it did not change the relationship between resources and development through the route of environmental improvement. (3) Export trade reduced the adverse impact of resource endowment on green growth and alleviated the severity of the resource curse phenomenon. However, export trade could only change the relationship between resources and development through environmental improvement, and not through energy conservation.

In conclusion, improving the import and export trade structure can reduce resource dependence to a certain degree; however, their roles are limited; the route to fundamentally alleviating the resource curse is through energy structure adjustment and green technological innovation. According to Vuong [45], investing in science, especially for research and development (R&D), will benefit society in the long run. China has invested a significant portion of its GDP in R&D, around 2.4 percent of GDP per year [46]; however, R&D investment in the ecological area still needs to be increased to improve energy structure and environmental quality. Additionally, in the context of the COVID-19 pandemic, promoting international trade in high-ecological technology value-added products is conducive to economic recovery and healthy economic development [47]. The Chinese government should take this as an opportunity, by shaping ecological values, promoting ecosurplus culture [48], and reducing the international trade of primary products, so as to reshape the innovative ecosystem, lessen the effects of the resource curse, and move toward a more sustainably green economy.

## Figures and Tables

**Table 1:** Definitions and descriptions of the variables.

Category	Symbol	Variables	Proxy Indicator
Explained variables	*tfp*	Green total factor productivity	Calculated by Equation (2)
	*tfp_e*	Energy conservation effect	Calculated by Equation (7)
	*tfp_u*	Environmental improvement effect	Calculated by Equation (8)
Explanatory variable	*re*	Resource endowment	Number of employees in the mining industry
Control variables	*import*	Import	Total import/Gross domestic product (GDP)
	*export*	Export	Total export/GDP
	*govern*	Environmental governance	Total investment in environmental pollution control/GDP
	*rd*	Research and development (R&D) investment	R&D capital stock/Gross domestic product
	*pergdp*	Economic development level	GDP per capita
	*indus*	Industrial structure	Secondary industry GDP/Total GDP
	*urban*	Urbanization level	Nonagricultural population/Total population
	*own*	Nationalization level	Number of employees in state-owned units/ Total number of employees

**Table 2:** Green total factor productivity and its decomposition results for various provinces.

Regions	Green Total Factor Productivity *tfp* (%)	Energy Conservation Effect *tfp_e* (%)	Environmental Improvement Effect *tfp_u* (%)	Regions	Green total Factor Productivity *tfp* (%)	Energy Conservation Effect *tfp_e* (%)	Environmental Improvement Effect *tfp_u* (%)
Beijing	7.783	1.411	6.372	Henan	−0.176	−0.237	0.061
Tianjin	0.583	−0.228	0.811	Hubei	0.365	−0.162	0.527
Hebei	−0.449	−0.518	0.069	Hunan	0.424	−0.289	0.713
Shanxi	0.058	0.084	−0.026	Guangdong	−0.032	−0.587	0.555
Inner Mongolia Mongolia	−0.381	−0.303	−0.079	Guangxi	−0.153	−0.791	0.638
Liaoning	−0.808	−0.587	−0.222	Hainan	−1.359	−1.285	−0.074
Jilin	0.330	0.215	0.115	Chongqing	0.296	−0.291	0.587
Heilongjiang	−1.756	−1.073	−0.683	Sichuan	0.100	−0.204	0.304
Shanghai	2.510	−0.698	3.208	Guizhou	0.743	0.465	0.278
Jiangsu	−0.367	−0.965	0.598	Yunnan	−0.638	−0.369	−0.269
Zhejiang	−0.707	−0.913	0.205	Shaanxi	0.087	−0.165	0.252
Anhui	−0.386	−0.517	0.130	Gansu	−0.240	−0.291	0.051
Fujian	−0.621	−1.159	0.538	Qinghai	−0.881	−0.497	−0.384
Jiangxi	−0.474	−0.735	0.261	Ningxia	0.276	−0.019	0.295
Shandong	−0.979	−0.702	−0.278	Xinjiang	−1.274	−0.865	−0.409
				Mean	0.062	−0.409	0.472

**Table 3:** Variable descriptive statistics.

Variables	Observations	Mean	Standard Deviation	Minimum	Minimum
Green total factor productivity (*tfp*)	338	−0.358	2.377	−12.391	7.010
Energy conservation effect (*tfp_e*)	338	−0.959	2.810	−15.879	7.746
Environmental improvement effect (*tfp_u*)	338	0.244	2.794	−10.774	10.576
Resource endowment (*re*)	338	20.485	20.455	0.471	103.014
Import (*import*)	338	11.079	12.005	0.417	72.594
Export (*export*)	338	13.338	16.937	0.728	92.927
Environmental governance (*govern*)	338	1.325	0.671	0.402	4.111
R&D investment (*rd*)	338	7.684	7.220	0.075	46.362
Economic development level (*pergdp*)	338	1.083	0.417	0.333	2.357
Industrial structure (*indus*)	338	47.526	6.942	22.327	61.478
Urbanization level (*urban*)	338	49.191	9.538	26.870	69.850
Nationalization level (*own*)	338	9.535	3.805	4.203	23.617

**Table 4:** Regression results of the fixed-effects model.

Variables	Model 1 (Full Sample)	Model 2 (Full Sample)	Model 3 (Full Sample)	Model 4 (Eastern)	Model 5 (Central)	Model 6 (Western)
	Green Total Factor Productivity (*tfp*)	Energy Conservation Effect (*tfp_e*)	Environmental Improvement Effect (*tfp_u*)	Green Total Factor Productivity (*tfp*)	Green Total Factor Productivity (*tfp*)	Green Total Factor Productivity (*tfp*)
Resource endowment (*re*)	−0.095 *** (−3.16)	−0.098 ** (−2.39)	−0.093 *** (−3.21)	0.151 ** (2.78)	−0.091 * (−2.37)	0.009 (0.10)
Import (*import*)	0.011 (0.17)	−0.031 (−0.54)	0.014 (0.18)	−0.052 (−0.46)	0.053 (0.29)	0.019 (0.37)
Export (*export*)	−0.072 (−1.01)	−0.025 (−0.40)	−0.119 (−1.47)	0.056 (0.43)	0.189 (1.62)	−0.186 ** (−2.32)
Environmental governance (*govern*)	−0.665 ** (−2.14)	−0.648 ** (−2.08)	−0.644 * (−1.93)	−0.498 (−1.21)	−1.161 (−1.36)	−0.564 * (−1.90)
R&D investment (*rd*)	0.121 (0.87)	0.147 (0.94)	0.057 (0.42)	0.711 * (2.16)	0.664 * (2.03)	0.058 (0.73)
Economic development level (*pergdp*)	3.018 (1.04)	4.074 (1.46)	1.039 (0.34)	6.705 * (2.08)	8.041 (1.76)	−2.927 (−1.55)
Industrial structure (*indus*)	0.042 (0.83)	−0.011 (−0.19)	0.111 ** (2.01)	0.347 ** (2.68)	−0.154 * (−2.10)	0.258 ** (3.09)
Urbanization level (*urban*)	0.194 * (1.72)	0.294 ** (2.50)	0.085 (0.71)	0.319 (1.18)	0.117 (0.61)	−0.002 (−0.01)
Nationalization level (*own*)	−0.244 (−1.30)	−0.212 (−1.14)	−0.250 (−1.17)	−0.484 (−1.76)	0.115 (0.41)	−0.001 (−0.00)
constant	Y	Y	Y	Y	Y	Y
year	Y	Y	Y	Y	Y	Y
province	Y	Y	Y	Y	Y	Y
Prob (F)	0.000	0.000	0.000	0.000	0.000	0.000
Observations	338	338	338	104	104	130

Notes: Robust t statistics aNotes: Robust t statistics are shown in parentheses. *** *p* < 0.01, ** *p* < 0.05, and * *p* < 0.1. The values in parentheses are T values. Y means yes, indicating that the constant term is included in the model, and the fixed effects for the year and province are controlled.

**Table 5:** Analysis of the import threshold effect.

Models	Threshold Type	F-Statistic	*p*	Critical Value		
				1%	5%	10%
Model 7 (*tfp*) Green total factor productivity	Single threshold	13.67 **	0.046	23.978	13.345	11.238
	Double threshold	8.98	0.132	53.122	19.254	10.539
Model 8 (*tfp_e*) Energy conservation effect	Single threshold	23.04 **	0.012	23.096	14.148	11.909
	Double threshold	10.35	0.158	90.519	58.523	20.983
Model 9 (*tfp_u*) Environmental improvement effect	Single threshold	4.73	0.620	16.913	13.163	10.895

Notes: Robust t statistics are shown in parentheses. ** *p* < 0.05.

**Table 6:** Estimated import threshold and confidence interval.

	Model 7 (*tfp*) Green Total Factor Productivity		Model 8 (*tfp_e*) Energy Conservation Effect		Model 9 (*tfp_u*) Environmental Improvement Effect	
	Estimated Value	95% Confidence Interval	Estimated Value	95% Confidence Interval	Estimated Value	95% Confidence Interval
Threshold γ	50.110	/47.632, 50.960/	50.110	/42.446, 50.960/	——	——

**Table 7:** Regression results of the threshold model with import as the threshold variable.

Variables	Model 7	Model 8
	Green Total Factor Productivity (*tfp*)	Energy Conservation Effect (*tfp_e*)
Resource endowment (*import* < 50.110) (*re_0*)	−0.084 *** (−2.72)	−0.043 (−1.24)
Resource endowment (*import* ≥ 50.110) (*re_1*)	−0.636 *** (−4.03)	−0.846 *** (−4.78)
Import (*import*)	−0.484 (−1.58)	−0.433 (−1.26)
Export (*export*)	0.073 (1.43)	0.095 * (1.66)
Environmental governance (*govern*)	−0.078 * (−1.64)	−0.056 (−1.10)
R&D investment (*rd*)	0.135 (1.18)	0.188 (1.46)
Economic development level (*pergdp*)	2.525 (1.53)	5.172 *** (2.80)
Industrial structure (*indus*)	0.136 *** (2.83)	0.146 *** (2.72)
Urbanization level (*urban*)	0.048 (0.75)	−0.004 (−0.05)
Nationalization level (*own*)	−0.220 * (−1.74)	−0.172 (−1.21)
constant	−8.250 *** (−2.82)	−11.844 *** (−3.61)
Prob (F)	0.000	0.000
observation	338	338

Notes: Robust t statistics are shown in parentheses. *** *p* < 0.01, and * *p* < 0.1. The values in parentheses are T values.

**Table 8:** Analysis of the export threshold effect.

Models	Threshold Type	F-Statistic	*p*	Critical Value		
				1%	5%	10%
Model 10(*tfp*) Green total factor productivity	Single threshold	11.64 *	0.068	17.065	12.264	10.293
	Double threshold	7.36	0.260	14.014	10.725	9.427
Model 11(*tfp_e*) Energy conservation effect	Single threshold	7.77	0.304	21.419	12.834	10.811
Model 12(*tfp_u*) Environmental improvement effect	Single threshold	9.06 *	0.096	13.446	10.381	9.025
	Double threshold	4.85	0.488	19.726	11.517	9.718

Notes: Robust t statistics are shown in parentheses. * *p* < 0.1.

**Table 9:** Estimated export threshold and confidence interval.

	Model 10 (*tfp*) Green Total Factor Productivity		Model 11 (*tfp_e*) Energy Conservation Effect		Model 12 (*tfp_u*) Environmental Improvement Effect	
	Estimated Value	95% Confidence Interval	Estimated Value	95% Confidence Interval	Estimated Value	95% Confidence Interval
Threshold γ	3.232	/2.781, 3.263/	——	——	3.076	/2.683, 3.141/

**Table 10:** Regression results of threshold model with export as the threshold variable.

Variables	Model 10	Model 12
	Green Total Factor Productivity (*tfp*)	Environmental Improvement Effect (*tfp_u*)
Resource endowment (*export* < 3.232) (*re_0*)	−0.184 *** (−4.24)	−0.233 *** (−4.40)
Resource endowment (*export* ≥ 3.232) (*re_1*)	−0.094 *** (−3.04)	−0.136 *** (−3.63)
Import (*import*)	−0.519 * (−1.70)	−0.506 (−1.37)
Export (*export*)	0.026 (0.52)	−0.012 (−0.20)
Environmental governance (*govern*)	−0.066 (−1.37)	−0.087 (−1.52)
R&D investment (*rd*)	0.124 (1.07)	0.060 (0.43)
Economic development level (*pergdp*)	4.167 ** (2.44)	0.777 (0.38)
Industrial structure (*indus*)	0.105 ** (2.14)	0.115 ** (1.94)
Urbanization level (*urban*)	0.015 (0.24)	0.073 (0.94)
Nationalization level (*own*)	−0.235 * (−1.85)	−0.300 * (−1.96)
constant	−6.005 ** (−2.01)	−2.286 (−0.63)
Prob (F)	0.000	0.000
observation	338	338

Notes: Robust t statistics are shown in parentheses. *** *p* < 0.01, ** *p* < 0.05, and * *p* < 0.1. The values in parentheses are T values.

## Data Availability

Publicly available dataset *China Statistical Yearbook 2018* was analyzed in this study. These data can be found at: https://data.stats.gov.cn accessed on 21 January 2022. Other raw/processed data required to reproduce these findings cannot be shared at this time, as the data also form part of an ongoing study.
